# Editorial for the Special Issue on Micro/Nano-Chip Electrokinetics, Volume II

**DOI:** 10.3390/mi9080383

**Published:** 2018-08-02

**Authors:** Xiangchun Xuan, Shizhi Qian

**Affiliations:** 1 Department of Mechanical Engineering, Clemson University, Clemson, SC 29634, USA; 2 Department of Mechanical and Aerospace Engineering, Old Dominion University, Norfolk, VA 23529, USA; SQian@odu.edu

There has been a rapidly increasing interest in the use of micro/nanofluidics to develop various point-of-care technologies for global health [1,2]. Electrokinetics is often the method of choice in these micro/nano-chips for an accurate transport and manipulation of fluids and samples [3,4]. This special issue in *Micromachines* is the continuation of our successful first volume on Micro/Nano-Chip Electrokinetics [5]. It consists of 22 contributions, which cover multiple aspects of electrokinetics related phenomena for various chemical and biological applications. We divide these papers into three primary categories and summarize them briefly below.

## 1. Linear Electrokinetic Phenomena (Seven Papers)

Fluid electroosmosis and particle electrophoresis are linear electrokinetic phenomena, and are proportional and parallel to the applied electric field [3,4]. Electroosmotic flow in micro/nanochannels can be significantly affected by the fluid rheological properties in addition to the wall properties. Guo and Qi [6] obtained an analytical solution of the electroosmotic peristaltic flow of viscoelastic fluids through a cylindrical microchannel using the fractional Jeffrey’s constitutive model. Choi et al. [7] carried out a finite element analysis of the electroosmotic flow of power-law fluids in a rectangular microchannel with asymmetric wall zeta potentials. They later [8] reported an approximate analytical solution to a similar flow in a slit microchannel. Mei et al. [9] studied the electroosmotic flow of a linear Phan–Thien–Tanner fluid in a nanoslit by solving numerically the nonlinear Poisson–Nernst–Planck equations. Matías et al. [10] presented a perturbation analysis of Joule heating effects on electroosmotic flow in a microcapillary tube filled with immiscible Newtonian and power-law fluids. Lu et al. [11] used a molecular dynamics simulation to study the electroosmotic flow in rough nanochannels, with particular attention to the fluid–solid interactions. Lim et al. [12] developed a technique to fabricate microchannels with black silicon nanostructures for a controllable suppression of electroosmotic flow.

## 2. Nonlinear Electrokinetic Phenomena (Eight Papers)

Nonlinear electrokinetic phenomena occur because of the action of electric field on an electrically induced dipole (i.e., dielectrophoresis or DEP) or free charge (e.g., induced charge electrokinetic phenomena and electrothermal flow). Akshay et al. [13] demonstrated significantly enhanced particle focusing and enrichment by the use of three-dimensional reservoir-based dielectrophoresis (rDEP) at the reservoir–microchannel junction. Zhao et al. [14] used a volumetric polarization and integration method to investigate the mechanisms for their observed tumbling motion of pearl chains and alignment of ellipsoidal particles. Both phenomena were found to be governed by the particle–particle interactions under DEP. Ji et al. [15] reported a direct numerical simulation of similar dielectrophoretic interactions between deformable particles. Liu et al. [16] proposed a method of bipolar field-effect control of direct current electroosmosis for multifunctional sample handling. Later, Tao et al. [17] used a similar idea to design nanofluidic ion diodes for field-effect control of ion current. Hu et al. [18] utilized the stirring fluid motion of induced charge electroosmotic flow over a floating gate electrode to improve the binding efficiency of microfluidic heterogeneous immunoassays. Ren et al. [19] explored the feasibility of using alternating current electric field-induced nonlinear electroosmosis next to sharp dielectric corners for on-chip mixing. Liu et al. [20] studied the electrode cooling effect on the travelling wave electrothermal flow in rotating electric fields.

## 3. Other Electric-Field-Mediated Phenomena (Seven Papers)

Song et al. [21] reported a numerical study of the electric-magnetic regulation of the heat convection between an electrolyte solution and microchannel walls for potential applications to microelectronics cooling. Shen et al. [22] demonstrated the use of optimized electrical driving waveforms to reduce the fringe phenomena in electrophoretic display. Zhang et al. [23] reported an on-chip impedance sensor that is capable of detecting both rigid particles and soft droplets/bubbles in hydraulic oil in an inductive and a capacitive mode, respectively. Yazdanshenas et al. [24] developed a microfluidic Kelvin water dropper that can generate high-voltage electricity through water dripping and also used their device to replace the high-voltage power supply in electrowetting. Hu et al. [25] demonstrated a microfluidic mixer design that utilizes amplified Marangoni chaotic advection induced by alternating current electrowetting of a metal droplet. Ahmed and Kim [26] presented a numerical parametric study of electroosmotic micro-mixers with heterogeneously charged surface patches on channel walls. In another study, Chen et al. [27] proposed and numerically verified the use of embedded asymmetric electrode arrays on microchannel walls to generate vortices for mixing enhancement.

We would like to express our sincere thanks to all of the contributors to this Special Issue. We also appreciate the time and efforts from all the reviewers, without which this Special issue would not be possible.
